# Managing the load on a reader’s mind

**DOI:** 10.1007/s40037-014-0144-x

**Published:** 2014-10-10

**Authors:** Jimmie Leppink

**Affiliations:** Maastricht University, Maastricht, Limburg the Netherlands

As Jan van Dalen opened in a previous issue [1], ‘It is every Editor’s dream that articles in “their” journal serve a wide population of interested readers.’ But how do we succeed in producing such articles?

What is of crucial importance is that an article provides answers to the questions ‘What is new?’ and ‘What is in there for me?’, and that it does not comprise too many elements that distract from these core answers. Cognitive load theory [2] provides a framework for how to do exactly that. In cognitive load theory, learning is the gradual development and refinement of schemata (i.e., existing knowledge) in long-term memory, and processing new information—information that is not yet part of these schemata—requires working memory load or *cognitive load* [3]. The extent to which an article presents new information depends on the level of expertise of the individual reader. For example, experts who have studied the human brain for quite some years tend to have very elaborate schemata of the human brain or at least of particular regions of the brain. Novice students, who have barely read or seen anything about the human brain, on the contrary, have virtually no schemata about the human brain or particular brain regions at all. If we wish to serve our entire reading audience, we need to take care that there is something new in our articles for both groups but that we do not overload the novice readers.

Explaining core assumptions and choices in sufficient detail is one way of managing this so-called *intrinsic cognitive load* [4] on the reader’s mind, providing references where readers can find more details and fill gaps in their knowledge is another way. If we fail to explain core assumptions and choices, or fail to provide accessible references on core issues about which we feel we cannot expand too much in our article (for instance, due to wording limits), we may overload potentially interested but novice readers, and we may lose their interest. Meanwhile, if we fail to provide meaningful answers to the questions ‘What is new?’ and ‘What is in there for us?’ we are unlikely to see experts in the field read our article, let alone use it for future research.

Apart from this so-called *intrinsic cognitive load*, which is defined by the amount of new information in the article (i.e., information that is not yet part of readers’ schemata), we may impose some *extraneous cognitive load* [5] on readers’ minds due to the way in which we present the information to our readers. For instance, suppose that—in a certain situation—a simple graph provides a clear explanation of particular findings. If we use lots of equations and jargon instead of such a graph, we require readers to process information that—in this particular situation—is not necessary for understanding the findings. Likewise, if we use large quantities of text to describe something like blood flow in the human body when a simple diagram can explain the same process equally well, having to process all that text seems *extraneous* to learning about the core process. An attractive and clear presentation of various parts of an article can help to minimize this redundant cognitive load and stimulate readers to deal with the intrinsic cognitive load (i.e., ‘What is new?’ and ‘What is in there for me?’).

In short, a good article has an interesting message that imposes some intrinsic cognitive load but as little as possible extraneous cognitive load on the reader’s mind. Something tells me that the articles in this issue have been accepted for publication exactly because they meet this criterion.
